# Imagined futures in living with multiple conditions: Positivity, relationality and hopelessness

**DOI:** 10.1016/j.socscimed.2017.12.022

**Published:** 2018-02

**Authors:** Lindsay-Ann Coyle, Sarah Atkinson

**Affiliations:** Durham University, Department of Geography and the Centre for Medical Humanities, Lower Mountjoy, DH1 3LE, United Kingdom

**Keywords:** Hopelessness, Multimorbidity, Narrative, Finance, Employment, Relationships, Care, Fairness

## Abstract

Hope serves as an overarching concept for a range of engagements that demonstrate the benefits of a positive outlook for coping with chronic conditions of ill-health and disability. A dominant engagement through medicine has positioned hope as a desirable attribute and its opposite, hopelessness, as pathological. In this engagement hope is individual, internally located and largely cognitive and able to be learned. Attaining hope reflects a process of coming to terms with the losses associated with long-term conditions and of imagining new meanings and purposes for the future ahead. This process is characterised by a set of linear temporal stages, from loss and denial to acceptance and reappraising the life-course, by an emphasis on the morally desirable exercise of self-care and by a desired outcome that, in the absence of cure, is hope. Through interviews, we aim to unsettle the privileged status given to a positive outlook through examining the expressions, contexts and negotiations of hopelessness of people living with multiple conditions of ill-health and/or disability. These narratives of hopelessness disclose the ways in which realistic imagined possibilities for the future are constrained by external structures of time and function that demand complex negotiations with places, bodies and other people. As a situated and relational narrative, hopelessness draws our attention to the need to rebalance the exclusive attention to individual, internal resources with a renewed attention to contexts and settings. Moreover, hopelessness can be generative for those living with multiple conditions in shaping alternatively framed priorities with respect to their temporal and interpersonal relations.

## Highlights

Unique contributions to knowledge:

• Narratives of hopelessness revealing challenges of living with multiple conditions.
• Consideration of different temporalities in narratives of hope and hopelessness.
• Critique of discourse of self-care for and by those living with chronic conditions.
• The generative potential of negativity through the example of hopelessness.

## 1 Introduction

It has become a truism that a positive outlook contributes to getting on in life and to managing adversity, including ill-health. The support for this comes from the strong and consistent association of positivity with various forms of coping and related self-reported psychological evaluations, such as self-esteem, self-worth and self-confidence. These relationships have been documented across a range of long-term conditions including those related to cancer, cardiovascular disease, respiratory failure, spinal cord injuries and ageing (Avvenuti et al., 2016, Livneh and Martz, 2014, Martz and Livneh, 2016). Whilst the corollary also holds that a lack of positivity is associated with poor coping, passivity and depression, research on the pathways underpinning these associations has largely privileged positivity operationalised through concepts such as optimism or hope. We have, by contrast, little insight into the emergence of hopelessness and its impacts, nor any consideration as to whether it is always undesirable or may ever constitute a resource for living with chronic conditions.

The paper aims to unsettle the privileged status given to a positive outlook through examining the expressions, contexts and negotiations of hopelessness of people living with multiple conditions of ill-health and/or disabilities (hereafter referred to as multiple conditions). The empirical data come from a larger qualitative study of the experiences of living with multiple conditions in which hopelessness emerged as a significant theme. We argue that medicine operates within a dominant cultural script comprising individualised temporal and linear stages of coming to terms with illness and disability and of imagining hopeful futures. The empirical data enable an interrogation of these narratives of time and hope through accounts by people living with multiple conditions about how they imagine their financial, health and relational futures.

Existing research on chronic ill-health or disability has predominantly related to cases of single, diagnosed conditions; there is, to date, only limited research in relation to living with multiple conditions. Research has also tended to be undertaken within medical settings rather than in the context of everyday lives (Ironside et al., 2003). Attending to the experiences of those living with multiple conditions is timely since their number is increasing rapidly. In the United Kingdom, this number will have increased by a million in just one decade, from 1.9 m in 2008 to a predicted 2.9 m in 2018 (Department of Health, 2012). Multiple conditions are recognised to present challenges to current medical practice and which recently have been addressed in the United Kingdom through new clinical guidance addressing issues such as the interactions of multiple drug prescriptions and the time demands of multiple symptoms on consultation scheduling (NICE(National Institute for Health and Care Excellence), 2016, Farmer et al., 2016).

## 2 Hope

Hope may be constructed in various ways: as a noun, inhering to objective circumstance or subjective resource, or as a verb, foregrounding the agency of the hoper and the act of hoping (Eliott and Olver, 2007). Hope is underpinned by diverse assumptions about where hope is located and how it may come to be: an entity or a psychosocial resource to acquire through having and maintaining hope (see Duggleby et al., 2012); an affective flow in being and becoming hopeful (see Anderson, 2006); a given disposition of personality through the binary of optimists and pessimists (see Carver et al., 2010); a moral virtue within traditional and contemporary expressions of spirituality and Christianity (see Crapanzano, 2003).

This diversity of engagements notwithstanding, the last thirty to forty years have witnessed the emergence within medicine of what is recognised as the current dominant understanding of hope in both scientific and popular thinking (see Eliott, 2005; who documents this). The development of ‘Hope Theory’ began in the 1980s, growing in close association with the expansion in psychology of the field of cognition and the turn to positive psychology. In this dominant understanding, hope is always good and desirable; it is located as an individual, internal and, mostly, cognitive perceived capability for identifying routes to desires and for motivating action to follow such routes. In this, hope may motivate the pursuit of both positive goals and the avoidance of negative outcomes (Snyder, 2002). Hope is one of a family of measurable constructs related to positivity that includes optimism, self-esteem, wellbeing and happiness (Alarcon et al., 2013, Eliott, 2005, Martz and Livneh, 2016, Snyder, 2002). The closest of these, optimism and hope, have been subtly differentiated as measurable constructs (Snyder, 2002) and as popular concepts have subtly different opposites (pessimism and hopelessness). Nonetheless, these nuances notwithstanding, hope and optimism also share a number of important attributes. First, they both concern positive thinking or imagination in relation to potential futures, and both treat such positivity as a good and desirable state. This is supported since both optimism and hope as constructs within positive psychology demonstrate consistency in direct and buffering effects on adapting well to chronic ill-health or disability, which is, in turn, associated with higher levels of positive self-worth, life satisfaction, quality of life and so forth (Martz and Livneh, 2016). Secondly, they are both positioned as internal to the individual and, perhaps most importantly, as states that can be learned, such that individuals can do something themselves or be helped to do something about their internal levels of hope or optimism (Seligman, 1991, Snyder, 2002). As such, both suggest that acquiring either hope or optimism become the responsibility of the individual, with the corollary that the fault for a lack of hope or optimism be similarly placed with the self. However, whilst these two concepts are closely connected, the term ‘optimism’ has become particularly associated with the positive psychology of Martin Seligman (1991) and, in part to countenance understandings beyond the dominant framing, we have favoured the less partisan language of hope and hopelessness as our overarching concepts.

Coming to terms with a chronic condition is often framed in terms of stages: as a staged grieving process (Dorsett, 2010) characterised by the expression of chronic sorrow (Ahlström, 2007) or stages of defiance and acceptance (Soundy et al., 2012). Defiance may be expressed both as initial denial but also as hope for stability in symptoms and retention of functions; acceptance may be expressed both through passivity and potential despair but also through reappraisal and finding alternative meanings and purposes for living (Soundy et al., 2012). In this framing, hope in relation to chronic ill-health or disability may always reflect a certain paradox in that finding hope, as an individualised, internal and desired goal, is closely connected to adjusting to the consequent losses in chronic ill-health or disability to bodily functions, relationships, an autonomous life, an expected life, roles, activities and identity (Ahlström, 2007, Soundy et al., 2012). Philosophers have offered an alternative framing in which understanding the processes of developing hopeful futures emphasises the role of the imagination within a multi-dimensional theory of emotions and interpersonal encounters (Simpson, 2004). Simpson draws on William Lynch's argument that hope, by definition, expresses an imaginative ability for identifying different future possibilities. While not all possible imaginings will be realistic or even necessarily positive, sharing one's hopes with others and the role of emotions as an interpretative framework serve to foreground those imaginings that have potential traction (Simpson, 2004). There are, of course, risks in imagining possibilities; the imagination can conjure negative, hopeless, as well as positive, hopeful, futures and thus the relationship between imagination and hope is not deterministic. Moreover, the dominant medical discourse of cure and progress may itself constrain the capacity to imagine beyond pre-defined ‘successful outcomes’ (Wendell, 1996).

The experiences of chronic illness and disability may undermine medicine's central ‘dominant cultural script’ (Dias, 2013: 31) in which the imagined future involves progress through treatment, remission and cure. However, positivity affords a way of repackaging this cultural script for those conditions with no cure and only likely deterioration. Combining stages to acceptance with imagining hopeful possibilities for the future effectively constitutes a parallel dominant script for medical approaches to chronic ill-health and disability: building psychosocial health as progress; acceptance as a variant on remission; hopeful imagined futures as an alternative desired outcome to cure. In a society that associates ‘getting well’ or ‘overcoming disability’ with progress, it is unpopular to think negatively about the already negative emotions associated with illness and disability such as pain, unhappiness and loss (see Atkinson and Rubinelli, 2012, Ehrenreich, 2009 in relation to cancer). This imperative to be hopeful is also associated with neoliberal economics, in which the promise of hope is similar to the promise of happiness and in which both are bought and sold (Davies, 2015, Good et al., 1990). Advancing the idea that everyone is entitled to happiness gains academic validation through the rise of positive psychology (Miller, 2008) and supports a marketplace for products through which this may be realised, such as self-help guides, material possessions and antidepressants (Schoch, 2006). But this narrative of hopefulness is not an automatic given, it is neither natural nor universal but is always intimately tied to particular notions of progress, morality and political ideology.

There are other less dominant elaborations of the notion of hope in circulation that also challenge some of the core tenets of the dominant biomedical construction. A primary assumption of positivity in general and hope in particular is that this is an unequivocally desirable state. The possibility of a profound challenge to the assumption of the desirability of hope is evident from historical studies of the very different social context of ancient Greece. Hope, for the Greeks and their adherence to an immutable destiny, was a highly ambivalent quality, more an evil than a virtue, and associated with illusion, confusion and folly (see Eliott, 2005). In contemporary engagements, biomedicine does appreciate that hope can sometimes have negative consequences if hope is misleading and prevents the individual facing difficult realities, particularly in relation to a poor prognosis in ill-health. This has mostly been framed as a bioethical question and related to the advocacy of full transparency in providing medical information to patients (Simpson, 2004), although Snyder (2002) also argues that the problem is overstated. However, ability to imagine and hope for what may seem impossible, the miracle last-minute cure, may be a vital part of coping for some patients that ought not be undermined (Dorsett, 2010).

A second core assumption is that hope is internal and an attribute of the individual. This again can be seen as a relatively contemporary understanding compared with a long history of hope within Judeo-Christian traditions, in which hope inheres in eternal rewards as a gift and promise from God and, as such, is transformative in the present through giving comfort and through prompting virtuous action (Eliott, 2005). Secularised variants of a virtuous hope similarly locate both its source and its promotion as external to the self. Hope is treated as resulting from inter-personal relations and directed to building a better society through movements for social reform (Marcel, 1978; Bloch, 1986; both in Eliott, 2005). This resonates with contemporary critical social and spatial theories of relationality, assemblage or affect in which experiential and cognitive concepts such as hope are seen as effects emerging from situations, relations and flows rather than as individually possessed attributes (see for example, Anderson, 2006, Atkinson, 2013, Delanda, 2016). Expressions of hope in medical settings are often taken at face-value and slotted into a linear frame that seeks to move the patient from despair to hope (see for example Smith and Sparkes, 2005). However, a more nuanced approach to the expression of hope focuses on how this is shaped by the situation, including the value given to being positive (Eliott and Olver, 2007, Good et al., 1990), and moves towards viewing hope as a ‘conversational idiom’ (Wilkinson and Kitzinger, 2000) and a ‘polyphonic narrative’ (Ezzy, 2000). In practical terms, viewing hope as relational, situated and polyphonic enables the patient, and those supporting them, greater flexibility in finding pathways, actions and diverse outcomes for hope beyond the primary medical focus of cure or coping (Ezzy, 2000).

Our research participants challenge the dominant conceptions of hopefulness. Those living with multiple conditions repeatedly experience ‘not fitting in’ to a wide range of social and narrative settings (Coyle, 2016): medical diagnostic categories; treatment regime; modes of employment, transport, housing and so forth. They do not even fit a meaningful category with each other since any constellation of symptoms and experiences is so particular. Listening to the voices of people expressing ‘bad’ emotions about the future is never easy (Wilkinson and Kitzinger, 2000), but this theme emerged frequently in our interviews and its exploration offers insights about the concepts of hope, time and futures under multiple conditions of ill-health and disability.

## 3 Research design

The data for this paper were generated as part of a qualitative study on the experiences more widely of living with multiple illnesses and/or disabilities. Participants were recruited through a mental health resource centre in the Northeast of England, The Waddington Street Centre. Those attending the centre live in the local area and are usually referred by the health or social services. The Centre provides life skills and opportunities for maintaining or improving mental health, including emotional support and courses on art, poetry, music, cooking and sports. Twelve participants were recruited from the Centre; three further participants who did not attend the Waddington Street Centre were recruited through a snowball sampling technique.

Potential participants were invited to a one-to-one semi-structured interview, a method that enables a compassionate and caring ambience in which people feel comfortable narrating their life experiences (Valentine, 2005). A list of topics was used to help prompt conversation (Valentine, 2005); for example, each participant was asked about certain topics such as ‘mobilities’ and ‘home’, which provided some structure to the interview but also enabled participants to narrate detailed personal accounts of their experiences.

The research design and methods were granted ethical approval through Durham University, whose procedures are compliant with the guidelines of the Research Councils of the United Kingdom (RCUK). In addition, we consulted the Centre staff on proposed practices throughout the period of the empirical research. The Waddington Street Centre stipulated their being named in any research outputs as a condition for support and entry to the research site which raised a particular ethical dilemma in relation to anonymity (Tilley and Woodthorpe, 2011); naming the Centre makes individual participants more easily identifiable, despite changing all personal names. This is doubly so in working with people living with multiple conditions since constellations of symptoms are often specific to an individual. Those interested in being interviewed first had a face-to-face discussion with the researcher about all aspects of the research process. Potential participants were made aware that it was not possible to guarantee anonymity, despite following best practice, given the Centre would be named. They then each completed a written consent form which had specified questions about the various aspects of the process and anonymity. In presenting participants’ experiences in this article, we are highly cautious about revealing the particular combination of illnesses and/or disabilities of any one person or other identifying characteristics, such as age, gender, race. This does lose the depth of insight afforded by individual identity characteristics of participants but was an ethical necessity.

Researchers embark upon any research endeavour with their own hopes for the process and their own pre-set ideas related to the central concepts that emerge. Andrews (2017) documents five types of hopes in the research process: specific hopes of the results; directional hopes of the field; imaginative hopes for a better future; hopes for the future wellbeing of participants; and hopes of reflecting and giving voice to the participants’ own hopes for their future. We recognise these underpinning hopes in our own research. As with most researchers in topics related to health, we carry with us hopes that the research will result in a betterment of circumstances for those struggling with multiple conditions. We also acknowledge our alignment with a growing number of scholars and commentators expressing unease with the uncritical privileging of various constructs of positivity across almost all domains of life and the industries accompanying this (examples of critiques include Burkeman, 2013, Davies, 2015, Ehrenreich, 2009, Whippman, 2016). As such, a part of our hopes involves contributing to this growing critique, exposing the logic of learned and internally generated positivity to justify victim-blaming, and to reaffirm the value of diversity in affect and cognition to include negativity (see Wilson, 2008).

Analysis of qualitative data necessitates choices about which themes are important, how to combine and interpret different participant voices and how to select extracts to illustrate the issues that emerge (Jackson and Mazzei, 2008, Moss and Dyck, 2003). We first pooled the interview transcripts and analysed them collectively in order to draw out broad themes that cut across the various individual experiences. We decided to use a conventional topic or domain focussed approach to indexing the transcripts (Atkinson and Abu El Haj, 1996, Silverman, 2014, Thomas, 2006) and through this process imagined futures and hopelessness emerged as important concerns from the participants' accounts. Each interview was also analysed as a single narrative so as to draw out the interplay between content and context in any one life-story (Wiles et al., 2005). The participants' accounts are taken at face-value; we have not engaged their accounts critically in order, for example, to draw out wider discourses and their influence on individual experience, perception or practice. Here, our primary interest is in the participants' own version of their experiences of living with multiple conditions, how they interpret the ways in which this intersects with various aspects of their daily lives and the challenges or opportunities they see their conditions presenting. The theme of imagined futures is broken down into three sub-topics of significance to participants: financial futures, health futures and relationship futures. The accounts in relation to these sub-topics are discussed in the next sections and, in keeping with standard practice, we draw on a small sample of our participants’ accounts to illustrate the issues in depth.

## 4 Findings

### 4.1 Financial futures

The implications of financial struggle are often excluded from research on hope and acceptance of long-term debilitating conditions. But poverty is a major form of disablement experienced by people whose bodies differ from societal norms and should not be underestimated:

“Poverty is the single most disabling social circumstance for people with disabilities, since it means that they can barely afford the things that are necessities for non- disabled people, much less the personal care, medicines, and technological aids they may need to live decent lives outside the institutions, or the training or education or transportation or clothing that might enable them to work or to participate more fully in public life.” (Wendell, 1996: 41)

Participants frequently articulated a sense of hopelessness about their financial prospects which needs to be understood in relation to other temporal modalities (Grosz, 2005).

Anna illustrates how the experience of her variabilities in bodily capacities often does not ‘fit into’ accepted distinctions made between ability/disability and health/illness:

*I don't actually get DLA [Disability Living Allowance: a government benefit] at the moment. Em, I'm just going onto Employment Support Allowance [another government benefit] but, obviously, the longer I'm on it, the more likely I am to have an assessment. And I think, because I'm actually physically able to walk and stuff like that, I think they are just going to say … ‘you're fit for work'. Em, but in my experience, when I've been this bad in the past, I'm not fit for work. I may have a few good days where I can go in, I can get on with my work. But all of a sudden I kind of almost flip out, you know? And it's like no can't do this no more. And I'm in tears and I'm getting angry and agitated and anxious … what employer wants that? Do you know what I mean? I mean, I know a lot of them are getting really good when it comes to disabilities and mental health and stuff like that. But at the same time, especially in today's job market, if you've got a choice between somebody with mental health issues and somebody who is perfectly well and, you know, fit and healthy, then they're going to take somebody who is fit and health really. They can afford to pick and choose. But the government doesn't seem to look at that side of things. You know, it's like, well if you can physically do this or you can physically do that then you can go to work. But they don't seem to take into account, you know, what it's like on a bad day.*

Anna is not hopeful about her present and future employment prospects since living with multiple conditions means being *uncertain* over how she may ‘fit into’ a work environment that assumes standardised, or at least predictable, bodily functions. Living with *uncertainty* about her bodily capacities provokes concern about the extent to which an employer would view her as an acceptable worker, particularly in a competitive labour market. This is compounded in that the uncertainty of her bodily variabilities are not easily categorised and, as such, make it difficult to accessing either employment or government benefits. On the one hand, Anna is concerned that those assessing benefit claims will not take into account ‘*what it’s like on a bad day’*; on the other, she is concerned that employers will *only* take into account what she is like on a bad day. This double-bind effectively results in exclusion from either route to accessing the necessary financial resources to maintain a decent standard of living.

The classification of people's experiences of bodily variabilities into ‘good days’ or ‘bad days’ is problematic (Lightman et al., 2009). Bodily variabilities cannot always be correlated with ‘good days’ and ‘bad days’, not least because bodies can change quickly and unpredictably, as Anna notes when she says that: *‘…all of a sudden I kind of almost flip out’*. Such uncertain bodily experiences make it difficult to ‘fit into’ the accepted temporal structures of modern life. Anna exemplifies this here in relation to the structure of the ‘day’, but which can extend to the ‘working week’ and ‘being on time’ which, again, can exclude access to both employment and government benefits. The narratives of hopelessness about financial futures by those living with multiple bodily variabilities are thus articulated within and through an awareness of the ‘double-bind’ they face.

The structures of employment are a central element in participants’ narratives of hopelessness in that particular workers and particular working styles are privileged at the expense of the physical, social and emotional inclusion of people whose bodies diverge from expected norms. Anna also explains that:

*…I left [my last job] because I hurt my back. I was working in a shop, so there was a lot of heavy lifting and stuff. But, yeah, I was in an office over in [name of place in the United Kingdom] and I wasn't technically employed. It was an employment training scheme, so it was a work situation. I was in a working office, em, but I actually did that. I was fine for a few months and then all of a sudden it was like the pressure just got to me, you know? Em, I don't know why. I suppose it was because I'd been there a while and I knew it wasn't going to last forever and I knew I was going to have to go and get a job. My anxiety just took over and one day I just flipped and I lost my temper with people around me and I got up and I just walked out and one of my supervisors came running down the street after me and she was like 'what on earth was all that about?'. And I just burst into floods of tears …*

Anna had to leave her first job in a shop after hurting her back, but the office environment of her second job was also difficult to manage because of her anxiety. It is unclear whether continuing to work in the shop made Anna's anxiety more manageable, but it is clear that the combination of both back and mental health problems influenced the types of environments in which she could work. So what was it about the structures of employment that Anna found difficult to negotiate?

Capitalist economic structures typically standardise tasks in relation to pre-existing conceptions of normal bodies, so that workplace practices become effectively disembodied to the disadvantage of those who do not ‘fit in’. For example, standard jobs include a broad range of tasks with an expected level of performance (Foster and Wass, 2013). First, this means that a person living with a condition that limits the ability to fulfil *one* aspect of the job description becomes unsuitable for the *entire* job. Secondly, the standardisation of work roles leaves the employment structures invisible and unchallenged through an apparent neutrality and objectivity, despite the resulting exclusion of particular bodies from the workplace. In the case of Anna, such standardisation may seem quite innocuous in the form of expectations that everyone can handle a certain amount of pressure and uncertainty related to working on fixed term contracts. However, Anna's particular conditions meant that whether she could or could not meet these expectations was both variable and uncertain across time. This is compounded by the constant change in expectations about what it is possible and reasonable to expect of bodies. In the United Kingdom, the financial crisis of 2008 brought austerity cuts in public sector funding including a rolling back of what constitutes ‘reasonable adjustments’ to working conditions so that people with disabilities may be employed (Harwood, 2014). These changes in employment structures leave people, such as Anna, sometimes unable to meet employers' requirements and, again, her account of negotiating work expectations is steeped in a sense of hopelessness about her present and future circumstances. Indeed, when Anna asks ‘*what employer wants that?*’ in relation to her being angry, agitated and in tears, she is effectively also asking who would employ someone with her multiply ill and disabled body.

The other side of the double-bind concerns accessing government financial assistance. In a third extract from the interview with Anna, she narrates the quandary she faces in negotiating the benefits application system:

*… when my doctor first gave me my sick note for my depression and anxiety, I know for a fact I should have taken it straight down to the dole [government benefits application office] and said 'I've got to go on the sick'. But I actually took a week out to consider whether I wanted to do it or not because I know what I'm going to face. I know I'm going to have to face my medical assessment and I know it means a change in benefits. So we've got to reapply for housing benefit and all that sort of stuff and the anxiety was, well, what happens if we miss a week's money? You know, if they're late sending us it we're going to be screwed for the rent and stuff like that. Em, and it does affect me really badly. In a way I would rather have stayed on job seekers' [allowance] because all the benefits were there, they were in place, they were claimed. But at the same time that means I've got to go and look for work which I know I'm not ready to take on, you know? So it's like, well what do I do? … so yeah, I do get very, very anxious about what's going to happen in the future but as a result of that I have to try and focus on today. I have to try and not let myself think about what's going to happen tomorrow because otherwise I wouldn't leave the house.*

Anna's reflections on whether to apply for government benefits are characterised by both uncertainty over what the future will hold and certainty that the future will be difficult. What-is-more, her certainty of future difficulty is based on previous experiences when she says, *‘I know what I'm going to face.*’ This is further exacerbated in changes to categories and thresholds for benefits, mirroring the changes in the workplace related to ‘reasonable’ adjustments (Harwood, 2014). For example, before the 2008 economic downturn, people could qualify for DLA (Disability Living Allowance) if their mobility was limited to under 200 m but this was later revised to under 20 m (Cross, 2013), representing a radical redrawing of the thresholds between ability/disability and health/illness in government benefits. It has become more difficult to be classified as ‘disabled enough’ to access available resources for support and describes a deepening of the double-bind of employment and benefits.

Anna thus illustrates a narrative of hopelessness that emerges from the financial implications of bodily variabilities, the structures of employment and the politics of accessing resources from the government together with changing thresholds in accessing support. The experiences for many people living with multiple conditions result in an imagined financial future in which life is not going to get better.

### 4.2 Health and care futures

Contemporary public health is characterised by the importance of self-care underpinned by an emphasis on individual responsibility as the primary impetus for preventing and managing illness (Fraser, 2004, Henwood et al., 2011). This individualist approach to public health in the context of people living with multiple conditions requires that people imagine a healthier, hopeful future as both desirable and achievable. Drawing on interviews with Mark, Vicky and Michael, this progressive emphasis is distant from the imaginations of health futures articulated by our research participants.

An important imperative in the self-care discourse of public health is the need to seek medical care as early as possible for new symptoms. In the context of negotiating multiple conditions, the possibility of ‘yet another problem to add to the list’ may not attract this normative urgency, as Mark's reflections on developing a new symptom illustrate:

*Now, recently I've been having problems down below…and I'd say about a month ago, this is how depression and anxiety can make you think, I was passing blood. Nothing else, [just] blood. And I didn't care … I was so fed up, stressed, depressed…I just thought: ‘if this is it; this is it’. I really, honestly, didn't care.*

This apathy towards a potentially serious medical problem in need of attention underscores the centrality of narratives of hopelessness to Mark's present and future imaginations of health. Mark does relate his apathy to his anxiety and depression, thus fitting the dominant cultural script, but his reluctance to engage with the medical system is also inseparable from his frequent, tiring and often frustrating experiences in previous consultations. Thus, the will and capacity to seek medical advice about the development of new symptoms is likely to be diminished for those already negotiating existing illnesses and/or disabilities. Vicky illustrates further the frustrations involved in accessing one or more diagnoses that will legitimate her ill-health and enable treatment (Moss and Dyck, 2003):

*I think that's really difficult as well, getting a diagnosis. I mean I've had maybe four or five, em, preliminary interviews or preliminary discussions with a new therapist who is going to pass me onto the next therapist, who'll pass me onto the right level of treatment. And every single time I've said, can you please give me a diagnosis, yes or no?*

Michael challenges the hopeful performance of self-care more explicitly in revealing an acceptance of his condition, a rejection of a hopeful future and a kind of defiance towards the self-care narrative:

*I have no, em, hopes or objectives as far as my life is concerned … I have no, I have no wishes for me and if somebody, eh, gave me a nice little cocktail one day to put me to sleep, I'd be perfectly happy with that. I'm not actively seeking, or have suicidal thoughts, but I'm getting a bit destructive in my own mind. And, em, I'm one of the few people who've started smoking again. Em, so what's the point? I mean, yeah it might give me cancer, but I'm going to die of something anyway so it doesn't really matter … the same applies to alcohol really, because alcohol isn't a very good fellow with the concoction of drugs that I take. But at the end of the day … I know it's being awfully selfish but if that were to cause liver failure or something, again, I've got to die of something. And I suppose it's my long suicide note. That I don't care. And I know it sounds awful because if I became ill that would involve other people looking after me and, that, I acknowledge and just say sorry.*

This explicit rejection of self-care through healthy behaviours as a ‘*long suicide note’* discloses little interest in longevity which, in turn, sits alongside a sense of hopelessness about every aspect of his imagined future. And yet, at the same time, Michael by his own account is not actively suicidal; he emerges from the account as mostly realistic and accepting of his unpromising and hopeless future.

A narrative of hopelessness is, thus, positioned as a significant feature of the imagined health futures of these participants. Those living with multiple conditions not only have to deal with present bodily differences and the difficulties accessing care and treatment. They also have to confront the temporalities of hope within dominant public health discourses in which medicine explicitly hopes to recover past health and maintain, or even improve, current health into the future. Both of these assume a universal hope for longevity and a healthy future expressed through the various models for the stages of hope recovery (see for example, Dorsett, 2010, Smith and Sparkes, 2005). However, our participants describe negotiating these temporalities through the experience and imagining of what are often health pasts, presents and futures characterised by hopelessness.

### 4.3 Relationship futures

Michael's quotation above intimates the ways in which his experiences of multiple conditions may impact on others who may have to look after him in some of his imagined futures. However, personal relationships go much deeper than simply relying on care provision from others. The process of imagining futures must often be negotiated with others, including close friends and family who may believe in and (re-)produce the dominant narrative of hopefulness and progress. This relational intertwining of imagined futures do not only relate to their own individual future lives, but extend to how they might cope with the change in their relationships with other closely-related bodies. It is the imagining of the futures of ‘other’ bodies and the implications of these futures on the participants' own bodies that compound the sense of hopelessness felt by many participants in this research.

‘Letting people down’ was a key theme in the narratives of imagined futures of several participants. Relationships with other bodies with different capacities, expectations and imagined futures were particularly difficult to negotiate. Patrick explained this in relation to planning holidays with his wife:

*… if my wife says we should really book a holiday this year … the first thought is, I would like to, but how am I going to be then [at the time the holiday is booked for]? I'm always unsure about the future … it's not just holidays, it's any decision. Big decisions. You think, I'd like to but I can't really commit myself fully to it because I don't know how I'm going to feel. It sort of feels not fair on my partner, to my wife, do you know what I mean? Cause I can't really say I'm going to be brilliant when we go. So it definitely influences my decisions for the future. I don't feel confident in making decisions because of my conditions.*

Patrick shows how his imagination of futures is tied to considering the implications of his unpredictable health for joint decision-making with his wife. Managing the variability in his health is not only about his own future bodily states, but also about how these may relate to his wife's future bodily states. In this instance, Patrick presents his future body as at odds with ‘being fair’ to his wife in an endeavour to mitigate the negative impacts of his health on his wife. In weighing up this issue of fairness, Patrick has decided that it is preferable not to book a holiday and not to make any big decisions rather than face the prospect of having to let his wife down if his health status changes. Connecting narratives of hopelessness to narratives of fairness underscores a tension that some participants in this research constantly juggle. For Patrick, it was more important to be fair to his partner than to hope himself, or to support his wife's hope, that going on holiday might be possible. Patrick's reasoning undermines a focus on the individual self as the primary locus for hope in several ways. Patrick's wife was evidently willing to hope and to provide the care and support needed to help Patrick take a holiday, stressing how the capacity for hope is often relational in vital ways. But Patrick himself also discloses how realising his hope for a holiday would have demanded that he failed to care or consider the effects on his wife if something were to go wrong. Again, this indicates the need to understand the capacity for hope and its opposite, hopelessness, as produced through relational and interpersonal processes. A potentially positive narrative of hopefulness is superseded by other narratives, in this case that of fairness, which unsettles dominant scripts of how people should think about the future. In Patrick's argument, hope is not necessarily either desirable or beneficial to the situation as he perceives it. At the same time, and in partial contradiction of this process of unsettling, Patrick's concern about ‘letting people down’ reflects a broader narrative that positions people living with multiple conditions as an inconvenience and devalues their different bodily form of human experience (Deal, 2007). These tensions between narratives of letting people down, being fair and being an inconvenience expose how a relational negotiation of imagined futures is a difficult and conflicted process for those living with multiple conditions.

Being fair to others is also an expression of caring for another. But relations of affection are entwined in complicated ways with how those living with multiple conditions may rely on one or two key people for emotional and physical support. Participants expressed considerable concern over the possibilities of losing important people in their lives. The love and support that certain family members and friends provided was often crucial to participants' ability to manage their multiple conditions. The possibility of such people themselves dying was very distressing to some participants, especially since many participants found it difficult to form and maintain new relationships. This entanglement of the research participants' imagined futures with the futures of other significant and loved bodies was part of how narrations of future illness and disability came to be imbued with a sense of hopelessness. For example, Angela's mother provides a great deal of support for her and she is concerned about how this might change in the future:

*My mum … supports me a lot. I couldn't manage without my mum.*

*I don't have any friends [pauses]*

*I don't manage well with relationships.*

[Later in the interview].

*When I look towards the future I generally tend to get myself into trouble and tend to get very depressed and very suicidal because I don't really see any good things in the future. I dream sometimes and come up with ideas of things I'd like to do. But realistically what I'd like to do is maintain my health so that I'm not in hospital, at least cope with basic, day-to- day things. And the future is very scary as well because I know that 1 day my mum might not be there.*

Angela's imagination of her future is intimately tied to the future of another, that of her mother. Her sense of hopelessness about the future is expressed through her concern that she ‘*couldn't manage without her mum’*. Parents very often fulfil important roles in caring and supporting adult disabled sons and daughters, but this role can be difficult to negotiate in the face of dominant expectations that adult children become independent of their parents (Shearn and Todd, 1997). Angela also noted that she does not have any friends and finds it hard to build relationships. Consequently, an important aspect of the hopelessness she feels is not only about the possible future death of her mother, but also about the absence of other current and future relationships. While Patrick's experience of hopelessness is partially tied to the idea of ‘letting people down’, Angela's is partially tied to the idea of being alone and the significance of absence in her future imaginations. Unpicking the presences and absences of relationality therefore reveals further ways in which narratives of futures are understood as hopeless. Participants appear caught in a further double-bind: on the one hand, trying to form and maintain relationships resulting in tensions around being fair to another; on the other hand, failing or not being able to cultivate such relationships resulting in the prospect of isolation and lack of support.

An important dimension of negotiating multiple conditions is therefore about living with the imagination that future relationships will be fraught with difficulty. This point is underscored by Angela's statement that when she thinks about the future she tends to ‘*get [herself] into trouble’*. Instead of imagining the future as happy and hopeful, she uses the language of being ‘realistic’ about her expectations and by just focussing on the present. Together with the importance of financial security and health, relationships are a third significant dimension to the narratives of hopelessness presented by participants in this research.

## 5 Discussion

We started the paper by describing a dominant cultural script for medicine's approach to chronic ill-health and disability. This is characterised by an emphasis on building psychosocial health by working through stages and processes of denial to acceptance and despair to hopeful futures. The benefits of positivity in the face of long-term conditions are well established and the aim of this paper is not to challenge the value of this in itself. Rather, our argument is that narratives of hopelessness deserve to be given equal attention in comprehending the experiences, logics and practices of those living with multiple long-term conditions. Through interviews with fifteen research participants living with multiple conditions, we identify three important narratives of hopelessness, each of which can be understood as emerging through a process of negotiation with various structures, previous experience and competing narratives. In this, the participants' narratives of hopelessness contrast the dominant engagements of hope in medicine and positive psychology in which this is predominantly presented as internal to the person themselves and, to an important extent, as a choice by the patient (Duggleby et al., 2012). While other voices are evident in the literature, and perhaps most importantly within the nursing literature that engage hope through relationality, polyphony, idiom and multiplicity (see for example Eliott and Olver, 2007, Ezzy, 2000, Penson, 2013, Wilkinson and Kitzinger, 2000), these remain in the shadow of the dominant approach. The emergence of a dominant narrative of psychosocial health as largely an internal product to be managed through intentional practices of positivity resonates well with public health's emphasis on individual responsibility in relation to our health-related choices. Whilst there is no doubt that programmes working with individuals suffering from depression and despair have important impacts through building more positive outlooks, the combined force of an internally focussed narrative from positive psychology and public health has built a dangerously one-sided view of the nature of hope and hopelessness.

Our participants' accounts of hopelessness challenge the dominant script of an individual internal positivity in three ways. First, they highlight that the narratives of hopelessness are multi-various; particular imaginations of participants' futures cannot be straight-forwardly understood as about only one aspect of life. Experiences of hopelessness are interlaced together in relation to several different aspects of their lives, including their concerns over their financial, health and relationship futures. Secondly, the logic expressed through participants' narratives of hopelessness relate explicitly to circumstances external to themselves. The financial insecurities of the past, the present and the imagined future are embedded within the structures of power at work in spaces of employment and welfare, through projected normative working bodies, thresholds of normality and disability, and shifts in these thresholds under different political and economic regimes. Similarly, an explicit rejection of the contemporary emphasis and expectation for self-care reflects our participants' awareness of the relative insignificance and futility of behavioural choices in the context of their wider health conditions. Finally, the need to take into consideration the imagined futures of those with whom our participants’ lives are intimately entwined, explicitly foregrounds how those living with multiple conditions must constantly negotiate and renegotiate the relationships, attitudes, settings and structures within which their everyday lives are embedded. In all three of these challenges, hopelessness may be viewed as generative in disclosing everyday hidden assumptions and implicit priorities. Hopelessness draws attention to the temporal and functional structures of the workplace, of the health and welfare sector, and the imagined progress of a life-course into which those living with multiple conditions find they do not fit. Perhaps most noteworthy is how attending to hopelessness negates the assumption that longevity is a universally desired hope. Instead, hopelessness engenders a different set of priorities in which consideration for the futures of others comes to the fore to privilege interpersonal virtues such as care or fairness.

The sense of hopelessness expressed by participants can, of course, be interpreted as itself another symptom of their conditions and, indeed, a number of the participants drew on the terminology of depression and anxieties. However, this narrow, medicalised understanding of people's imagined futures serves to negate, silence or diminish the relational and situated experiences of our participants. Although depression, stress, anxieties and so forth were often elements in the constellations of multiple conditions, the participants were not strictly in denial about their conditions nor had they not accepted their situation. Rather, they engaged their circumstances through their own experiential knowledge and within a broad scope of external processes. Moreover, they rarely viewed their lives in terms of a linear progression from a past stage of limited insight towards a more successful stage in the future. That said, the narratives of hopelessness presented by participants do conform in one regard to a linear form of time. Their imagined futures were either as bad or worse than the present, reflecting the dominant linear understanding of time and deterioration (Milojević, 2008). Hence, although the participants' imagined futures typically destabilise hegemonic conceptions of time that privilege progress, morality and accumulation, this is complicated by a reproduction of society's dominant linear conception of time in relation to deterioration (see Thomson, 2005).

In conclusion, the paper demonstrates the need to listen more attentively to the voices of people living with multiple conditions and not to dismiss or pathologise their imagined futures simply because they challenge a dominant critical script for futures imagined as mostly hopeful. An attention to the ways in which those living with multiple conditions may constructively imagine their futures as hopeless discloses the central importance in their experiences of wider structures, assumptions and expectations that foreclose realistic imagined possibilities. For this growing group of people, the ubiquitous exhortation to individual responsibility for self-care in order to achieve positive thinking and hope systematically obscures important dimensions affecting the everyday experience of negotiating how to live with multiple conditions.
